# Ultrasonographic diagnosis of clinical and subclinical bovine respiratory disease in Holstein calves

**DOI:** 10.14202/vetworld.2022.1932-1942

**Published:** 2022-08-17

**Authors:** Ahmed E. Mahmoud, Ahmed Fathy, Eman Abdelhakim Ahmed, Asmaa O. Ali, Ahmed M. Abdelaal, Mamdouh M. El-Maghraby

**Affiliations:** 1 Department of Animal Medicine, Faculty of Veterinary Medicine, Suez Canal University, Ismailia, Egypt; 2 Department of Animal Wealth Development, Biostatistics Division, Faculty of Veterinary Medicine, Suez Canal University, Ismailia, Egypt; 3 Department of Animal Medicine, Faculty of Veterinary Medicine, Zagazig University, Sharkia, Egypt

**Keywords:** bovine respiratory disease, calves, haptoglobin, thoracic ultrasound

## Abstract

**Background and Aim:**

Bovine respiratory disease (BRD) is the main cause of death in calves, and early BRD diagnosis saves lives. This study aimed to diagnose clinical and subclinical BRD in calves by assessing some biochemical alterations and ultrasonography (USG).

**Materials and Methods:**

Fifty-four Holstein dairy calves in Al-Sharqiyah Province, Egypt, were used in the study. They were divided into three groups. The first control group consisted of 10 clinically healthy calves. The second group consisted of 34 calves suffering from clinical lower respiratory tract disorders. The third group consisted of 10 subclinical BRD-affected calves. Ultrasonographic examinations of chest and thoracic ultrasound scoring were performed once per 2 weeks for each calf. Blood samples were collected for serum separation to measure albumin (ALB), total protein (TP), ALB, globulin, and haptoglobin (HP).

**Results:**

The USG revealed small consolidation areas within an aerated lung lobe, a hypoechoic parenchyma of the entire distal lung lobe, and a hypoechoic-circumscribed structure surrounded by an echogenic wall appeared within the lung tissue in calves that suffered from lobular pneumonia, lobar pneumonia, and lung abscess, respectively. However, subclinical cases showed a small consolidation area in the cranial aspects of the right cranial lung lobe. The ultrasound lung score (ULS) was greater in clinical than in subclinical cases. The BRD-affected calves recorded significant increases in serum TP, globulin, and HP. Meanwhile, serum ALB decreased significantly.

**Conclusion:**

Thoracic ultrasound had a reliable tool in the BRD diagnosis, especially in the early prediction of subclinical cases in newborn calves. In addition, the ULS appeared to be a better classifier than the clinical respiratory score (CRS) for BRD diagnosis. On the other side, it was found that regression models were very useful in assessing the prediction of biochemical blood parameters based on the ULS and CRS in diseased cases.

## Introduction

One of the most common health problems in dairy calves is a bovine respiratory disease (BRD) [1]. It is a complex inflammatory disease caused by pathogenic and opportunistic viruses and bacteria in association with adverse environmental conditions and calves’ immunity impairment [2]. Fever, anorexia, nasal and ocular discharges, coughing, varying degrees of breathing difficulties and loudness, fast breathing, drooping ears, open-mouthed breathing, and death are the most prevalent BRD symptoms [3].

The BRD complex is predominantly associated with young calves, heifers, and steers starting from the 1^st^ week of life [4]. Furthermore, Wisnieski *et al*. [5] reported that BRD in dairy calves and its consequences were associated with increased rearing costs, increased mortality or relapse risks, and impaired growth and early culling. Moreover, BRD resulted in 23% of deaths during the pre-weaning period. Therefore, BRD has a serious economic impact on farms [6].

The BRD induced serious loss in the farm economy due to an increased mortality rate, especially in pre-weaned calves [6], and increased treatment and rearing costs as reported by Wisnieski *et al*. [5]. Hence, early BRD detection is one of the challenges for veterinarians and researchers [7].

Serum biochemical abnormalities were widespread in BRD-affected calves, and they seemed to be rather predictable changes in response to inflammation, resulting in considerable variations in protein profiles [8]. In addition, thoracic ultrasonography(TUSG) is considered a non-invasive diagnostic tool that has been used for lung pathology parenchyma assessment, pleural effusion detection and characterization, pulmonary consolidation, and pneumothorax [9]. In addition, TUSG gives an immediate result in contrast to radiographs [7]. Ultrasound lung score (ULS) is a scoring system that is performed to assess lung infection severity and ranges from 0 to 5 based on the mass in five lung lobes [6].

Clinicians’ treatment decisions for patients are based on diagnostic and screening tests’ capacity to detect disease existence or severity. The diagnostic sensitivity (SE, i.e., probability of a positive test outcome in a diseased individual) and specificity (SP, i.e., probability of a negative test outcome in a non-diseased individual) can be estimated by comparing the dichotomized test results to the true status of individuals that were determined by “gold standard” test as mentioned by Greiner and Gardner [10]. Regression models are intended to aid healthcare professionals and patients in making decisions about diagnostic tests, therapy initiation and discontinuation, and lifestyle changes [11].

This study aimed to assess (1) the TUSG use for diagnosing clinically BRD-affected calves and early subclinical cases detection, (2) BRD diagnostic test evaluation using receiver operating characteristic (ROC) curve, and (3) multiple models application for assessing the relationship between clinical, ULS, and biochemical parameters in clinical and subclinical BRD cases.

## Materials and Methods

### Ethical approval

The Scientific Research Ethics Committee for Animal Research at the Faculty of Veterinary Medicine, Suez Canal University, Egypt, authorized all procedures utilized in this study (No. 2021027).

### Study period and location

The study was carried out from December 2020 to March 2021 with a predominating temperature of up to 13°C (8–22), relative humidity of up to 30% (26–35), and average rainfall according to the geographical location of all of the study areas. This study was conducted on 54 Holstein dairy calves from 30 days of age until weaning (2 months) that is reared in Al-Salhia Farm in Al-Sharqiyah Province, Egypt.

### Animals and management

The calves were reared in an artificial outdoor rearing system in which the calves are separated from their dams after 6 h of birth to allow them to suck colostrum. Each calf was housed in a separate pen for 2 weeks and then removed to a collective alternative hutch system (8–10 calves in each wire) to facilitate the cleaning and scratching of the dirty floor. The floors were disinfected from time to time using slaked lime in the animals’ presence and/or absence without stimulating the dustiness to minimize respiratory disease development.

Calves were born normally from a vaccinated dam against the infectious bovine rhinotracheitis virus, parainfluenza 3 virus, bovine respiratory syncytial virus, and bovine viral diarrhea virus. As recommended by the Association of Official Analytical Chemists International, calves were given three meals of milk and calf starter at a rate of 2–3 L/meal/calf [12]. In addition, tap water was accessible as needed.

Calves were classified into three groups according to clinical respiratory score (CRS) according to Love *et al*. [13]. The score is based on rectal temperature, characteristics of nasal and ocular discharges, presence of cough, and ear position, as shown in Table-1. This score is the sum of the points obtained from these clinical signs that ranged from 0 to 15. A high score indicates the progressive severity of respiratory diseases. Calves with scores of ≥4 had at least three clinical signs, are considered diseased, and are included in the study. Conversely, those with CRS ≤3 are considered normal calves.

**Table-1:** Clinical respiratory scoring chart.

Degree	Criteria			

	0 (normal)	1 (mild)	2 (moderate)	3 (severe)
Temperature	37.7–38.2	38.3–38.8	38.8–39.3	≥39.3
Nasal discharge	Scanty serous discharge	Small amount of unilateral cloudy discharge	Bilateral cloudy or excessive mucous	Copious bilateral mucopurulent
Ocular discharge	No discharge	Small amount of ocular discharge	Moderate bilateral ocular discharge	Copious ocular discharge
Cough	No cough	Induced single	Induced repeated or occasional spontaneous	Repeated spontaneous
Ear and head position	Normal	Ear flick or head shake	Slight unilateral droop	Head tilt or bilateral droop

Calves were classified into three groups as follows: The first was the control group (G_1_, n = 10), which were clinically and apparently healthy and did not show any pulmonary lesions by TUSG. The second group (G_2_, n = 34) suffered from clinical lower respiratory tract disorders and had pulmonary lesions by TUSG, which was considered as the clinical BRD group. Furthermore, the third group (G_3_, n = 10) was clinically and apparently healthy and had pulmonary lesions by TUSG, which is categorized as the subclinical BRD group.

### Clinical and USG examination of animals

All the newborn calves (n = 54) were subjected to the clinical examination of the respiratory system as described by Nagy *et al*. [14]. Moreover, TUSG examinations were applied once per 2 weeks using a portable linear transducer, as cited by Ollivett and Buczinski [15]. The USG investigations started dorsally on the right calf at the level of the scapula in the sixth intercostal space (ICS) and progressed cranially to the right first ICS. This instrument is used to examine the right middle lung lobe as well as the caudal and cranial aspects of the right cranial lung lobe. On the left side, the USG examination started dorsally at the scapula level in the left sixth ICS and progressed cranially to the left second ICS. This is to examine the caudal and cranial aspects of the left cranial lung lobe while scanning the right and left caudal lung lobes from the 6^th^ to 10^th^ ICS.

**Figure-1:**
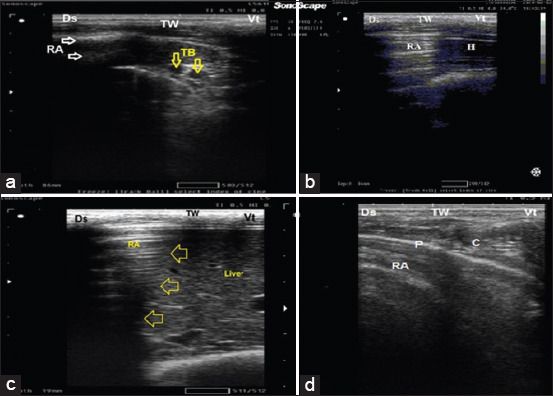
Thoracic ultrasonographic image of control calves. (a) Ultrasonographic of cranial aspect of cranial lobe of the right lung with reverberation artifact and thoracic blood vessels (artery and vein) linear probe is placed on 2–3 intercostal space (ICS). (b) Ultrasonographic of caudal aspect of cranial lobe of the right lung with reverberation artifact (dorsally) and heart (ventrally), linear probe was placed on 3–4 ICS. (c) Ultrasonographic of caudal lobe of the right lung with hepatic parenchyma appeared as hypoechoic structure with an echoic blood vessel (ventrally) and lung appeared with normal reverberation artifact (dorsally), the line of separation (yellow arrow) represents diaphragm, linear probe was placed on 6–10 ICS. (d) Ultrasonographic of middle lobe of the right lung with reverberation artifact and costochondral junction, linear probe is placed on 5–6 ICS. Ds=Dorsal, Vt=Ventral, RA=Reverberation artifact (white arrow), TW=Thoracic wall, TB=Thoracic blood vessels, yellow arrow=Diaphragm, H=Heart, and P=Pleural line.

The probe was placed within each ICS parallel to the ribs and moved ventrally toward the costal arch or the sternum until specified USG landmarks were visualized and then cranially up to the right first or left second ICS to examine the cranial aspect of the cranial lung lobe as described by Ollivett *et al*. [16] (Table-2 and Figure-1).

**Table-2:** Landmarks for the right and left lung lobes during ultrasonographic examination.

Lung lobes	ICS	Ventral landmarks
Right		
Cranial		
Cranial aspect of cranial lobe	1–2	Internal thoracic artery and vein
Caudal aspect of cranial lobe	3–4	Heart
Middle	5	CCJ and pleural deviation
Caudal	6–10	Diaphragm and liver
Left		
Cranial		
Cranial aspect of cranial lobe	2–3	Heart
Caudal aspect of cranial lobe	4–5	CCJ and pleural deviation
Caudal	6–10	Diaphragm and liver

ICS=Intercostal space, CCJ=Costochondral junction

### Ultrasound lung score

The ULS was carried out in five lung lobes for each calf, according to Cramer and Ollivett [6] as shown in Table-3.

**Table-3:** Ultrasound scoring chart.

TUS	Description	Definition
0	Normal or <1 cm^2^ consolidation	Normal
1	Diffuse comet tails	Normal
2	Consolidation ≥1 cm^2^	Lobular pneumonia
3	1 entire lung lobe consolidated	Lobar pneumonia
4	2 entire lung lobes consolidated	Lobar pneumonia
5	≥3 entire lung lobes consolidated	Lobar pneumonia

TUS=Thoracic ultrasound scoring

### Sampling

A 3 mL blood sample was collected from each calve along the study (n = 54) in a plain tube, allowed for clotting for 60 min, and then centrifuged at 1107× *g* for 20 min to isolate sera stored at −20°C for biochemical analysis [17].

### Biochemical analysis

Total protein (TP) and albumin (ALB) concentrations were determined calorimetrically using commercial kits (Spectrum Co., Egypt). Meanwhile, globulin concentration was estimated as the difference between TP and ALB. Consequently, the A/G ratio was calculated by dividing the ALB value over the globulin value according to Fischbach and Dunning [18].

Serum levels of haptoglobin (HP) were assessed using enzyme-linked immunoassay kits (Life Diagnostics Co., USA) according to the method described by Alsemgeest *et al*. [19].

### Statistical analysis

Data were statistically analyzed using a one-way analysis of variance with SPSS software version 20 (IBM Corp., NY, USA). The obtained data were shown as mean ± standard error of the mean. p = 0.05 was used to determine significance.

#### Diagnostic test performance using ROC curve

The odds of detecting proper diagnosis by test among true diseased subjects (D+) and true non-diseased subjects (D−) are two popular markers of a medical test’s inherent statistical validity (D−). For dichotomous responses, a 2 × 2 contingency table, also known as a confusion matrix, can be used to describe the results in terms of test positive (T+) or test negative (T−). The rows indicate the test results, whereas the columns show the binary categories of true sick status. The gold standard is used to determine true status [20], as illustrated in Table-4.

**Table-4:** Diagnostic test results in relation to true disease status showed in 2*2 confusion matrix.

Diagnostic test results	True disease status	

	Present	Absent
Present	TP	FP
Absent	FN	TN

The numbers along the major diagonal denote correct decisions, whereas the numbers along the off-diagonal denote errors (confusion between various classes). TP=True positive, FP=False positive, FN=False negative, TN=True negative

Some equations of several common metrics that can be calculated from a confusion matrix;

1. SE (also called true-positive rate, hit rate, and recall)


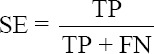


2. The false-positive rate or false alarm rate


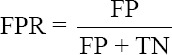


3. SP


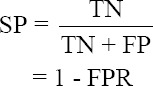



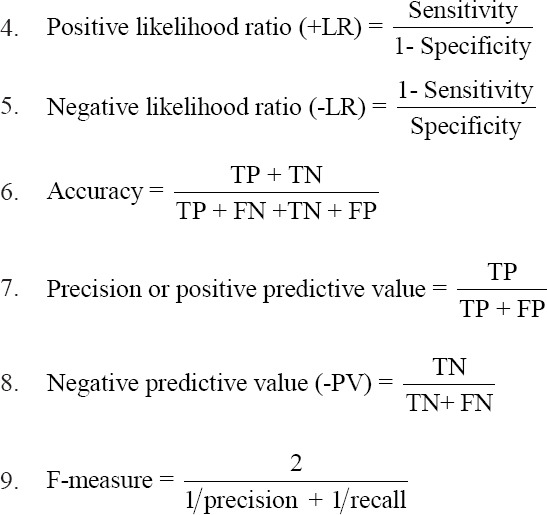


Equations 1, 4, 5, and 6 called performance measures [21].

#### Non-parametric approach for calculating the area under the curve (AUC)

For determining the AUC, statistical software provides both parametric and non-parametric methods. The non-parametric technique (Mann–Whitney U-test) is the ideal choice in this study because it does not require any distribution pattern for the test values and the resulting AUC is referred to as empirical. The first method employs the trapezoidal rule. It estimates the area by simply merging the points (1-SP, SM) at each interval of the continuous test observed values and drawing a straight line connecting the X and Y-axis [20].

#### Methods for the selection of the optimum cutoff value or the threshold point

The best point cut is one that ensures the most accurate classification. The first approach involves measuring the distance between the point (0,1) and any point on the curve (d_2_ = [1 − SE]_2_ + [1 − SP]_2_). The ideal cutoff point, which displays the best discriminating point between sick and non-diseased instances, is the one with the shortest distance. The second method is the use of Youden index J, which is the farthest point from the line of equality (diagonal) and indicates the maximum (SE + SP). It may be found by measuring the vertical distance between the equality line and any point on the curve, with the farthest position being the best. The second method is the most commonly used one [22].

#### Multivariate regression

In multivariate regression, calculations were made for variation estimation caused by the independent variables on the dependent variable concurrently [23]. The multivariate regression analysis was formulated as follows:

*y = β_0_+ β_1_ S_ULS_* + *β_2_ S_CRS_* + *ε*

Where,

y = Observed value of the dependent variables

β_0 =_ Constant

β_1-2_ = Unstandardised Regression Coeffient for each predictor variable

ε = Error

Predictor variables:

*S_ULS_*: Score of ultrasound lung score

*S_CRS_*: Score of clinical respiratory signs

The assumptions of multivariate regression analysis, such as normality, linearity, multicollinearity, missing, and extreme values [24], were tested first using IBM SPSS^®^ 26.00 [25] and then used to develop a multivariate regression model.

The Kolmogorov–Smirnov test was used to check the normality for residuals. The Mahalanobis distance and Chi-square test were used to determine the presence of outliers. Using a basic scatter plot, the data were also checked for linearity between the outcome and explanatory variables [26]. Finally, the variance inflation factor was used to test multicollinearity among ostensible discriminators [27].

All statistical analyses were carried out using the SPSS V. 26.0 [25] and MedCalc software version 20.100 (MedCalc^®^ statistical software, Chicago, Illinois, USA).

## Results

### Clinical findings

The clinical BRD group showed pronounced respiratory manifestations compared to the control and subclinical cases. In addition, CRS of the clinical BRD cases showed a significant increase when compared with the control and subclinical cases, respectively (Table-5).

**Table-5:** The main clinical findings and the mean values ± SE of CRS in comparisons among the different groups.

Criteria	Groups		

	Control cases (G1) (n = 10)	Clinical cases (G2) (n = 34)	Subclinical cases (G3) (n = 10)
Clinical findings			
Temperature	38.8^b^ ± 0.21°C	40.5^a^ ± 0.25°C	38.3^b^ ± 0.42°C
Nasal discharge	Scanty serous discharge	Bilateral mucopurulent	Small amount of serous discharge
Ocular discharge	No discharge	Bilateral mucopurulent	No discharge
Cough	No cough	Moist paroxysmal	Induced cough
Dyspnea	Not present	Present	Not present
Auscultation of trachea	Bronchial sound	Rattling sound	Exaggerated Bronchial sound
Auscultation of chest	Vesicular sound	Coarse crackles	Vesicular sound
CRS	2.2^b^ ± 0.18	6.02^a^ ± 0.19	2.6^b^ ± 0.31

Means carrying different superscripts in the same row are significantly different at p *≤* 0.05. CRS=Clinical respiratory score, SE=Standard error

### Thoracic USG and ULS

Ultrasound examinations of the lung of apparently healthy calves (G1) were characterized by a hyperechoic line with reverberation artifact and indicative of the normal pleural interface (Figure-1). Based on TUSG examination, the clinically BRD-affected calves (n = 34) were subdivided into three subgroups as follows: Calves lobular pneumonia (n = 22), lobar pneumonia (n = 9), and lung abscess (n = 3).

**Figure-2:**
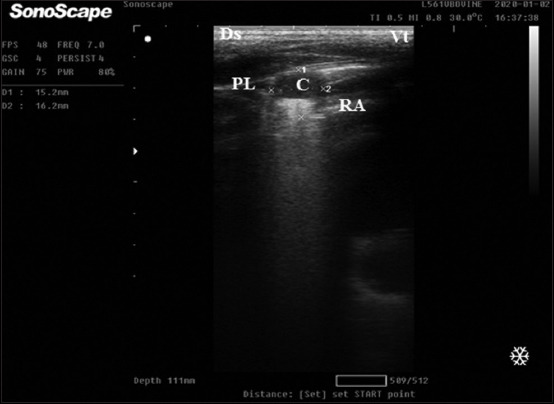
Thoracic ultrasonographic image of clinical bovine respiratory disease calves affected by lobular pneumonia. Examination of lung showed multiple areas of consolidation (1.5 × 1.6 cm) in ventral of caudal aspect of cranial lobe of the right lung linear probe placed on 3–4 intercostal space with reverberation artifact. Ds=Dorsal, Vt=Ventral, PL=Pleural line, RA=Reverberation artifact, and C=Consolidation.

**Figure-3:**
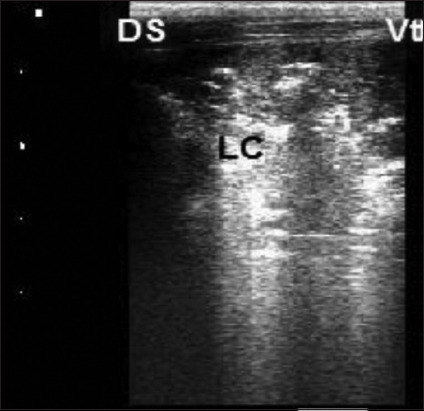
Thoracic ultrasonographic image of clinical bovine respiratory disease calves affected by lobar pneumonia. Examination of lung revealed hyperechoic structure (lung consolidation) of all cranial lobe of the left lung, linear probe placed on 2–5 intercostal space. Ds=Dorsal, Vt=Ventral, and LC=Consolidation.

**Figure-4:**
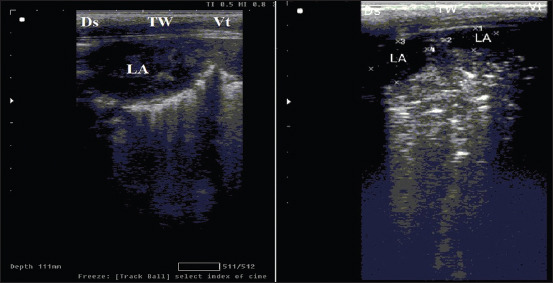
Thoracic ultrasound of clinical bovine respiratory disease calves affected by lung abscess. Examination of lung showed hypoechoic circumscribed structure surrounded by echogenic wall appeared within lung tissues. Right lobe; multiple small-sized abscess in cranial lobe of the left lung sized 1 × 2 cm, linear probe placed on 2–3 intercostal space (ICS). Left lobe; single large abscess in cranial lobe of the right lung sized 3 × 4 cm, linear probe placed on 1–4 ICS. Ds=Dorsal, Vt=Ventral, LA=Lung abscess, and TW=Thoracic wall.

**Figure-5:**
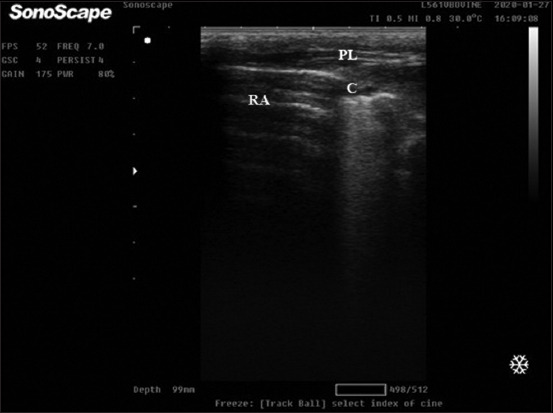
Thoracic ultrasound of subclinical bovine respiratory disease-affected calves. Examination of cranial aspect of cranial lung lobe of the right lung revealed of small area of consolidation (0.5 cm), linear probe placed on 1–2 intercostal space. PL=Pleural line, RA=Reverberation artifact, and C=Consolidation.

Calves that suffer from lobular pneumonia are characterized by relatively small discreet areas of consolidation within aerated lung lobe. In other words, the hyperechoic pleural interface with reverberation artifact of the normal lung can be seen dorsal and ventral to the lobular lesions (Figure-2). However, calves with lobar pneumonia are characterized by full-thickness consolidation of the lung lobe that extends proximally from the tip of the lobe. In the ultrasound image, the hypoechoic parenchyma of the entire distal lung lobe is visible, and the aerated lung cannot be seen ventral to the lesion (Figure-3). Moreover, calves with lung abscesses are characterized by either hypoechoic or anechoic circumscribed structures surrounded by an echogenic wall that appears within lung tissue may be single or multiple (Figure-4). However, the ultrasound examination of subclinical cases of 2-week-old calves showed small areas of consolidation (0.5 cm in diameter) that is noticed in the cranial aspect of the right cranial lung lobe (Figure-5). Table-6 summarizes the ULS of different groups. The ULS in the case of lobar pneumonia and lung abscess showed a significant increase compared to lobular pneumonia, subclinical BRD cases, and the control group. Meanwhile, lobular pneumonia and subclinical cases showed a non-significant change compared to the control group.

**Table-6:** The mean values ± SE of ULS of BRD-affected calves compared with control group.

Parameter	Groups					p-value

	Control	Lobar	Lobular	Abscess	Subclinical	
ULS	0.60^c^ ± 0.24	4.20^a^ ± 0.20	2.20^b^ ± 0.20	4.60^a^ ± 0.24	1.60^b^ ± 0.24	<0.0001

Means carrying different superscripts in the same row are significantly different at p *≤* 0.05. SE=Standard error, ULS=Ultrasound lung score, BRD=Bovine respiratory disease

### Comparative evaluation for the classification performance of both ULS and CRS using the ROC curve

**Figure-6:**
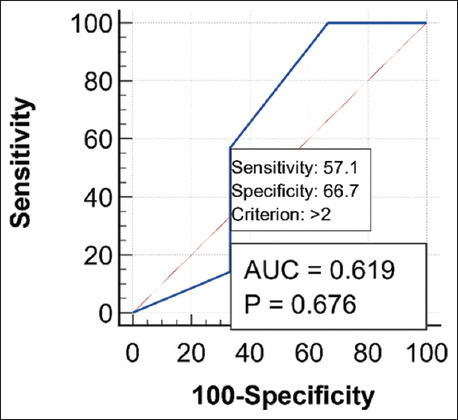
Receiver operating characteristic curve for clinical respiratory signs.

**Figure-7:**
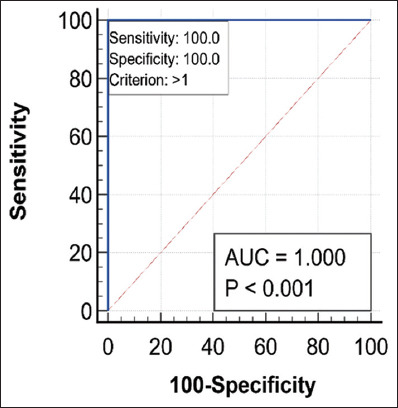
Receiver operating characteristic curve for ultrasound lung score.

**Figure-8:**
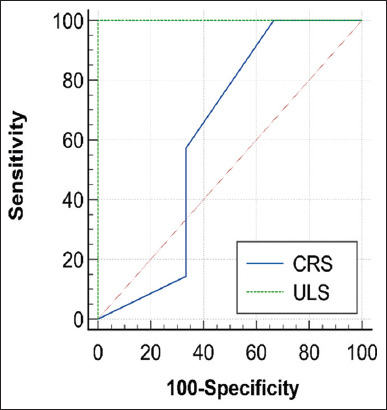
Receiver operating characteristic curve comparison for both ultrasound lung score and clinical respiratory signs.

**Figure-9:**
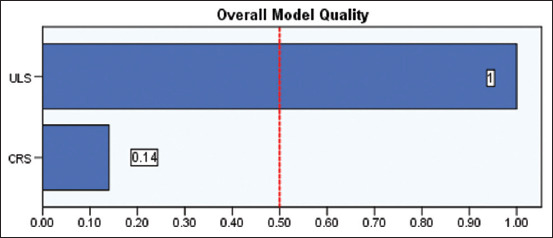
A good model has a value above 0.5. A value < 0.5 indicates that the model is no better than random prediction.

Comparisons of the diagnostic performance for both ULS and CRS in differentiating normal and subclinical BRD cases that were done using the ROC curve, the AUC, standard error, 95% confidence interval, and significance tests for CRS and ULS diagnostic tests are revealed in Table-7. The ROC curve for ULS was found to pass through the upper left corner (100% SE and 100% SP) with an AUC of 1.00. Conversely, AUC for CRS equals 0.619. Figures-6 and 7 show the AUC for both CRS and ULS, separately. Meanwhile, Figure-8 shows the AUC for both CRS and ULS. It was obvious that the ULS performance was better than the CRS performance. Table-8 demonstrates the optimum criterion value associated with the maximum correct classification for ULS and CRS. The optimal cutoff point for ULS was >1 with associated coordinates (100% SE and 100% SP). The optimum threshold for CRS was >2 with associated coordinates (57.14% SE and 66.67% SP). Table-9 represents the performance measures, including precision, recall, and F1 score. The performance measures for CRS were 33.33% accuracy, 80% precision, 57.14% recall, and 87.5% F1max. The performance measures for the classifier ULS were 100% accuracy, 70% precision, 100% recall, and 100% F1max. The overall model quality is shown in Figure-9 and reveals that a good model has a value above 0.5. A value <0.5 indicated that the model is not better than random prediction. Therefore, ULS is a good model and could depend on when comparing diseased and non-diseased cases with subclinical BRD cases.

**Table-7:** Area under the ROC curve, SE, 95% CI, and significance tests for CRS and ULS diagnostic tests.

Diagnostic test	AUC	SE	95% CI (AUC)	Z statistic	p-value (AUC=0.5)
CRS	0.619	0.285	0.278–0.890	0.418	0.6759
ULS	1.00	0.000	0.692–1.00	-	<0.0001

ROC=Receiver operating characteristic, AUC=Area under the curve, SE=Standard error, ULS=Ultrasound lung score, CI=Confidence interval, CRS=Clinical respiratory score

**Table-8:** Criterion value (cutoff value) and associated coordinates of the ROC curve of the studied diagnostic tests (CRS and ULS).

Diagnostic test	Criterion	Sensitivity	Specificity	+LR	-LR	+PV	-PV
CRS	>2	57.14	66.67	1.71	0.64	16	93.3
ULS	>1	100.00	100.00	-	-	100.00	100.00

*+LR=Positive likelihood ratio, -LR=Negative likelihood ratio, +PV=Positive predictive value, -PV=Negative predictive value, ROC=Receiver operating characteristic, CRS=Clinical respiratory score, ULS=Ultrasound lung score

**Table-9:** Performance measures of the classification models for both diagnostic tests (CRS and ULS).

Diagnostic test	Youden index J (accuracy)	Precision	Recall	F1 max
CRS	33.33	80	57.14	87.50
ULS	100.00	70	100.00	100.00

CRS=Clinical respiratory score, ULS=Ultrasound lung score

### Biochemical analysis

Clinical BRD cases showed significant (p ≤ 0.05) elevations in the mean values of serum TP, globulin, and HP compared to subclinical and control healthy cases. Meanwhile, significant (p ≤ 0.05) decreases in the mean values of serum ALB and A/G ratio were recorded in clinical cases compared to the other groups. Conversely, non-significant (p > 0.05) differences were noticed between the subgroups of clinical BRD cases regarding the biochemical parameters (Table-10).

**Table-10:** The mean values ± SE of serum levels of proteins, albumin, globulin, A/G ratio, and haptoglobin of BRD-affected calves compared with the control group.

Parameters	Groups				

	Control cases (G1) (n = 10)	Clinical cases (G2) (n = 34)			Subclinical cases (G3) (n = 10)

		Lobular pneumonia (n = 22)	Lobar pneumonia (n = 9)	Lung abscess (n = 3)	
Total proteins	5.68^b^ ± 0.09	6.91^a^ ± 0.13	7.63^a^ ± 0.13	7.42^a^ ± 0.13	5.72^b^ ± 0.80
Albumin	2.87^a^ ± 0.02	1.96^b^ ± 0.11	2.10^b^ ± 0.11	2.36^b^ ± 0.11	2.95^a^ ± 0.16
Globulin	2.81^b^ ± 0.08	4.94^a^ ± 0.16	5.53^a^ ± 0.16	5.06^a^ ± 0.16	2.77^b^ ± 0.66
A/G ratio	1.02^a^ ± 0.02	0.39^b^ ± 0.03	0.37^b^ ± 0.03	0.47^b^ ± 0.03	1.15^a^ ± 0.19
Haptoglobin	32.14^b^ ± 1.6	40.13^a^ ± 2.24	40.13^a^ ± 2.24	40.13^a^ ± 2.24	27.44^b^ ± 1.36

Means carrying different superscripts in the same raw are significantly different at p *≤* 0.05. SE=Standard error, BRD=Bovine respiratory disease, A/G ratio=Albumin/globulin ratio

### Development of prediction models for biochemical blood parameters based on ULS and CRS

Multiple linear regression was carried out to develop a regression model for the prediction of biochemical blood parameters TP, ALB, globulin, A/G ratio, and HP based on the ULS and CRS scores as predictors. First, the assumptions were tested to make sure if they were failed to comply with or not. It was found that the Kolmogorov–Smirnov test for residuals ranged between 0.072 and 0.104 with P = 0.200, which enhances the violation of normality that does not exist. The absence of multivariate outliers was shown by Mahalanobis distance (minimum Mahalanobis = 0.087, maximum Mahalanobis = 8.28, Chi-square test = 9.21, and p > 0.01). Scatter plots that were used to test the linearity assumption revealed a linear relationship between the dependent variables and each predictor variable. The multicollinearity diagnosis results showed that the tolerance was 0.573 which was not less than 0.2 and cannot cause any multicollinearity problem. The variance inflation factor of 1.745, which lies between 1 and 5, indicated that the correlation between predictor variables was moderate and ensured by the simple linear correlation (0.654).

Multiple linear regression models for forecasting biochemical blood parameters based on the information are shown in Table-11, as follows:

*y_TP_*=5.41+0.18 *S_ULS_*+0.13 *S_CRS_*

*y_ALB_*=3.15−0.18 *S_ULS_*+0.015 *S_CRS_*

*y_G_*=2.26+0.36 *S_ULS_*+0.12 *S_CRS_*

*y_A/G ratio_*=1.23−0.113 *S_ULS_*−0.025 *S_CRS_*

*y_HP_*=27.24+1.78 *S_ULS_*+0.35 *S_CRS_*

**Table-11:** Multiple linear regression predictive model summary for biochemical blood parameters.

Model	R	R^2^	Adjusted R^2^	Standard error of the estimate	Change statistics				

					R^2^ change	F change	df_1_	df_2_	Sig. F change
TP	0.633	0.401	0.377	0.643	0.401	17.053	2	51	<0.001
ALB	0.765	0.586	0.569	0.307	0.586	36.038	2	51	<0.001
G	0.736	0.542	0.524	0.810	0.542	30.126	2	51	<0.001
A/G ratio	0.749	0.561	0.543	0.233	0.561	32.534	2	51	<0.001
HP	0.775	0.600	0.584	3.35042	0.600	38.263	2	51	<0.001

TP=Total protein, ALB=Albumin, A/G ratio=Albumin/globulin ratio, G=Globulin, HP=Haptoglobin

The coefficient of determination determined the goodness of fit. Parameter values for the goodness of fit are mentioned in Table-12. The R^2^ value was the largest between the predictor variables and HP as a biochemical blood parameter by 0.600. The correlation between the predictor variables and HP was 60%, and the smallest correlation was observed for TP by 40.1% (R^2^ = 0.401).

**Table-12:** Multiple linear regression coefficient for prediction of biochemical blood parameters.

Predictors	Biochemical blood parameters	Unstandardized coefficients		Standardized coefficients	t	Sig.
	
		β	S.E	Beta		
Constant	TP	5.41	0.216	-	24.96	<0.001
	ALB	3.15	0.104	-	30.35	<0.001
	G	2.26	0.273	-	8.269	<0.001
	A/G ratio	1.23	0.079	-	15.659	<0.001
	HP	27.24	1.129	-	24.131	<0.001
CRS	TP	0.13	0.056	0.466	3.257	0.002
	ALB	0.015	0.027	−0.793	−6.658	<0.001
	G	0.12	0.071	0.640	5.110	<0.001
	A/G ratio	−0.025	0.20	−0.679	−5.539	<0.001
	HP	0.35	0.294	0.711	6.081	<0.001
ULS	TP	0.18	0.085	0.221	1.541	0.129
	ALB	−0.18	0.04	0.043	0.364	0.718
	G	0.36	0.107	0.136	1.084	0.284
	A/G ratio	−0.113	0.031	−0.100	−0.819	0.416
	HP	1.78	0.441	0.092	0.786	0.435

CRS=Clinical respiratory score, ULS=Ultrasound lung score, TP=Total protein, ALB=Albumin, A/G ratio=Albumin/globulin ratio, G=Globulin, HP=Haptoglobin

## Discussion

The BRD complex represents a medical issue in veterinary medicine due to the difficulty of clinical diagnosis, exacerbated by a dearth of gold standard diagnostic assays [28]. The TUSG was used to examine the pathological lesions associated with lung affections and was shown to be a reliable method for assessing lung consolidation and the degree of lesions in calves; moreover, it may be conducted conveniently in a field setting [7]. In this vein, the study was designed to elucidate the most common lesions among BRD-affected calves through thoracic ultrasound scoring (TUS) and determination of some biochemical parameters in the blood that could assist the early BRD prediction.

In the present study, the major clinical signs of BRD-affected calves observed were consistent with those observed by Metwally *et al*. [29] and Ramadan *et al*. [30]. The observed nasal discharge and coughing might be attributed to irritation and inflammation of mucous membranes, and inspiratory dyspnea might attribute to severe inflammation in bronchioles and alveoli that interfere with gas exchange and respiration. In addition, abnormal tracheal and lung sounds were ascribed to the presence of exudates that were produced by goblet cells as a sequence of inflammation [31].

The TUSG allows assessment of the extent and severity of pulmonary changes and seems to be suitable as a screening tool for the detection of pathologic lung changes near the pleura as they can be recognized if lesions begin at the thoracic wall or the diaphragm or extend toward the lung surface. Moreover, evaluation of the deep parts of the lung is not possible if the superficial part of the lung is normal due to air presence between the probe and the lesion [32].

Misdiagnosis of BRD lesion had been recorded by Ollivett *et al*. [16] who stated that researchers did not routinely and systematically extend their examinations cranially beyond the third ICS. Therefore, an ultrasonographic examination must be extended from the right first and second ICS as BRD lesions often start in the cranial aspect of the right cranial lung lobe [16].

Pulmonary consolidation can be localized, or it could impact the entire ventral part of a pulmonary lobe. The extent of pneumonia can be evaluated by visualizing either small localized lesions or lesions that involved the entire part of a pulmonary lobe [9].

In clinical and subclinical BRD-affected cases, the cellular infiltrates and cellular debris displaced air from the lung tissue resulting in non-aerated and/or consolidated lung lesions. The consolidated lung appeared as homogenous hypoechoic structure that resembles the liver in USG imaging [33].

Lesions of lung abscess corresponded to alveoli that are filled with either liquid or a more cellular material or consolidated pulmonary lobules. Furthermore, these abscesses are observed only if they are located against the visceral pleura [9].

The first consolidation episode appeared at the 1^st^–3^rd^ weeks of age (1 and 3 cm cutoff), respectively. The main cause of this lesion might be the rapid mixing of the newly born calves with a large number of older calves from the 1^st^ week to 3 months of age in the pre-weaning pens, as mentioned by Abdallah *et al*. [34].

The systematic TUS was used in the present study to examine the 3^rd^–11^th^ ICS and to determine if lung consolidation is ≥1 cm. In addition, ULS can be able to recognize the differences between normal aerated lung, aerated lung with diffuse pleural roughening (also called comet tail artifact), lobular lung consolidations, and lobar lung consolidations [15].

Fawcett [35] mentioned that the AUC is an important statistical way of evaluating the classifier’s performance, as it corresponds to the probability that the classifier could select a positive instance higher than the randomly selected negative one. The ULS classifier is better than the CRS classifier as it is the perfect classifier with 100% SE and 100% SP. A diagnostic test with perfect differentiation between diseased and non-diseased groups has a maximum AUC = 1. This confirms that SE and SP are 1, and both errors (false positive and false negative) are 0 [20].

The performance measures for ULS were higher than that of CRS, so ULS had a perfect discrimination power over CRS, which is not a realistic classifier in differentiation between normal and subclinical BRD cases. In addition, the AUC, used as a tool in evaluating the classification power of a diagnostic test, precision, and recall, is common in evaluating the overall performance of a diagnostic test [36, 37].

Assessment of serum TP assessment is a reliable indicator for passive transfer of immunity from dam to calves that are essential for keeping the health status [38]. In the present study, clinical BRD calves revealed a significant increase in serum TP and globulin. These findings are in accordance with Šoltésová *et al*. [39], who attributed the increase in the level of positive acute-phase protein (TP and globulin) during the infection to face the demand of the animal body for amino acids for hepatic protein synthesis.

Decreased ALB values observed in pneumonic calves concur with the earlier findings stated by Civelek *et al*. [40] that may be allied with possible hepatic dysfunction with a subsequently decreased liver ALB synthesis persuaded by inflammatory responses. Conversely, increased globulin levels in BRD-affected calves may be attributed to the severity of inflammation that requires increasing the levels of immunoglobulin, especially γ-globulin [41].

Under field conditions, serum HP is considered the most sensitive acute-phase protein for early diagnosis of calves with the highest risk of BRD and in monitoring the treatment efficacy [42]. A highly significant increase in mean values of HP could be explained by the elevated serum HP level in BRD-affected calves, indicating a strong immune response to lung affections, which results in cell injury of lung tissues. Moreover, serum HP is considered a strong bacteriostatic protein against various bacterial pathogens by binding with free hemoglobin and denying microorganisms from iron required for their development [8].

## Conclusion

It can be concluded that TUSG has a very important role in the early prediction of subclinical BRD in newborn calves. In addition, ULS appeared to be a better classifier than CRS, which is a non-realistic classifier. Alternatively, it was found that regression models were very useful in assessing the prediction of biochemical blood parameters based on ULS and CRS scores. Moreover, HP concentration is considered a reliable indicator of respiratory tract affection in newly born calves.

## Authors’ Contributions

AEM: Clinical examination, collected samples, analyzed the data, and drafted the manuscript. AF: Statistical analysis and made discussion for the results. EAA: Ultrasonographic examination and drafted the manuscript. AOA: Clinical examination, data analysis, and drafted the manuscript. AMA: Ultrasonographic examination and revised the manuscript. MME: Designed the experiment and drafted and reviewed the manuscript. All authors have read and approved the final manuscript.
